# Bovine Digital Dermatitis: *Treponema* spp. on trimming equipment and chutes – effect of washing and disinfection

**DOI:** 10.1186/s12917-024-03941-z

**Published:** 2024-06-18

**Authors:** Lina Ahlén, Ingrid Hunter Holmøy, Åse Margrethe Sogstad, Tim Kåre Jensen, Sara Frosth, Anna Rosander, Terje Fjeldaas

**Affiliations:** 1 Department of Production Animal Clinical Sciences, Faculty of Veterinary Medicine, Norwegian University of Life Sciences, P.O. Box 5003, Ås, 1432 Norway; 2 Animalia, P.O. Box 396, Økern, Oslo, 0513 Norway; 3 Department of Veterinary and Animal Sciences, University of Copenhagen, Ridebanevej 3, Frederiksberg C, 1870 Denmark; 4 Department of Biomedical Sciences and Veterinary Public Health, Faculty of Veterinary Medicine and Animal Science, Swedish University of Agricultural Sciences, P.O. Box 7036, Uppsala, SE- 750 07 Sweden

**Keywords:** Claw trimming, Swabs, Biopsies, qPCR, FISH, Passive transfer

## Abstract

**Background:**

Digital dermatitis (DD) is a contagious bovine foot disease causing reduced animal welfare and negative economic consequences for the farmer. *Treponema* spp. are the most important causative agents. Studies indicate that trimming equipment can transfer DD-associated treponemes between cows. The aim of this observational study in 22 DD-positive Norwegian dairy herds was to investigate the risk of transferring *Treponema* spp. with trimming equipment and chutes after claw trimming, and after washing and disinfection. Swabs from the trimming equipment and chutes were collected from nine different locations, at five different time points. Bacterial DNA was extracted from 647 swabs and analysed by qPCR for *Treponema* spp. In addition, 172 swabs taken immediately after trimming, were analysed by a multiplex qPCR targeting *T. phagedenis*, *T. pedis* and *T. medium/vincentii.* Biopsy sampling from DD lesions was performed on cows in the same herds during trimming. Altogether 109 biopsies were analysed by FISH for confirmation of the DD diagnosis and identification of *Treponema* phylotypes (PTs).

**Results:**

High numbers of *Treponema* spp. were detected from all nine locations on the trimming equipment and chutes immediately after trimming, and *T. phagedenis* was detected on two or more locations in all but two herds, 1 and 19. There was a decline in the amount of *Treponema* spp. after washing and disinfection. The belly belt, the cuff, and the footrest on the chute had the highest proportion of positive samples after disinfection. The belly belt had the highest copy numbers of all nine locations (median = 7.9, max = 545.1). No *Treponema* spp. was detected on the hoof knives after disinfection. *Treponema phagedenis, T. pedis*, and *Treponema* phylotype 3 (*T. refringens*) were detected by FISH analysis of the biopsies. *Treponema phagedenis* was detected in biopsies from all herds except 1 and 19.

**Conclusion:**

This study shows that DD-associated *Treponema* spp. were present on the trimming equipment and chutes after trimming cows in DD-positive herds. Washing and disinfection reduced the load of *Treponema* spp. However, large differences in *Treponema* spp. between different locations were documented. High copy numbers on the grinder and the chute after disinfection, indicates that sufficient cleaning and disinfection of these locations is difficult, and that passive transfer of DD-associated treponemes (viable or not) is possible.

**Supplementary Information:**

The online version contains supplementary material available at 10.1186/s12917-024-03941-z.

## Introduction

Digital dermatitis (DD) is a contagious bovine foot disease with negative impact on production and animal welfare. The disease is described as an infection of the digital and/or interdigital skin with erosions, mostly painful ulcerations and/or chronic hyperkeratosis/proliferation [1]. Even though the exact aetiology and pathogenesis of the disease is not determined, there is agreement that different infectious agents, the immunity and genetics of the host, and environmental factors are involved [2]. Multiple studies have demonstrated the presence of a variety of bacteria [3–5]. However, there is agreement and substantial evidence that *Treponema* spp. are the most important causative agents [6–8]. The phylogroups *Treponema medium/Treponema vincentii*, *Treponema phagedenis* or *Treponema pedis* have consistently been detected within the DD lesions [9–12]. The dissemination of the disease has increased rapidly during the last 10–20 years, and purchase of live animals is an important risk factor [13]. Ahlén et al. [14] found an association between increasing number of purchased animals during a period of five years and the odds for a herd to be DD positive.

Frequent routine claw trimming has been associated with DD [14–16] and studies have demonstrated that there is a potential for passive transfer of the disease between animals within herds with the trimming equipment [17, 18]. Sullivan et al. found that treponemes from at least one of the three phylogroups associated with DD were present in at least 42% of swabs from hoof knives used to trim cattle with DD lesions [17]. In another study, Bell (2017) identified DD-treponema DNA, positive for both the phylogroups *T. phagedenis* and *T. medium*, on the hoof grinder disc and the handle after trimming [19].

Professional claw trimmers visit many different herds weekly, sometimes daily. In Norway they prefer to use their own trimming equipment and chute to work safely and efficiently, which may imply a potential risk of transferring DD between herds. Already in 1999 Wells et al. documented that lack of washing of trimming equipment was associated with high within-herd incidence (> 5%) of DD (OR = 1.9) due to passive transmission of the disease [20]. Another study on contagious ovine digital dermatitis (CODD) has shown that DD-associated *Treponema* spp. can survive on rubber gloves for three days [21].

The importance of controlling the spread of the disease is emphasized by the limited scientific evidence regarding the efficiency of treatment of DD [2]. A study by Krull et al. indicated a high rate of DD-lesion recrudescence following a single tetracycline treatment (44%) when followed for an extended period of time (679 d) [22]. After introduction in a herd, the disease is difficult to eliminate. Alongside with controlled exchange of animals between herds, proper washing and disinfection of trimming equipment and chutes are considered essential to limit the spread of DD, and previous studies have designed different protocols to optimize these procedures [23, 24]. Hartshorn et al. (2013) developed a protocol for the in vitro minimum bactericidal concentration of various foot bath disinfectants for a *T. phagedenis*-like microorganism where manure contamination, potentially resulting in inhibition of the effect of the disinfectant, was mimicked [23]. Later Staton et al. (2020) developed a disinfection protocol for hoof knives to be used during claw trimming. The removal of visible dirt in a bucket with water and soap before submerging the knives in the disinfectant solution (FAM®30, 2% Virkon® or sodium hypochlorite) for 20 s is essential in the protocol [24]. However, the most important tools needed for routine claw trimming are the grinder and the trimming chute. Our hypothesis is that higher numbers of *Treponema* spp. will be found on the grinder and the chute compared to the hoof knife because they are more difficult to wash and disinfect. If so, the grinder and the chute could potentially be more important for the dissemination of DD than the hoof knife.

Our aim was to investigate DD-associated *Treponema* spp. on trimming equipment and chutes after routine claw trimming in DD-positive dairy herds. We also aimed to investigate the effect of washing and disinfection on the amount of *Treponema* spp. on the same equipment and chutes.

## Materials and methods

### Study design

This observational study compared the load of *Treponema* spp. on different trimming equipment and chutes at routine claw trimming in 22 Norwegian dairy herds with DD, using quantitative polymerase chain reaction (qPCR). In addition, a multiplex qPCR targeting *T. phagedenis*, *T. pedis,* and *T. medium/vincentii* was included. The diagnosis of DD in these herds was confirmed by identification of *Treponema* spp. in biopsies from DD lesions by fluorescent in situ hybridization (FISH).

### Selection of trimmers and herds

Data from the Norwegian Dairy Herd Recording System (NDHRS) was used to select herds and claw trimmers. The NDHRS is a nationwide recording system owned by farmers. It contains information on cow pedigree, production, and health of individual animals in enrolled herds owned by farmers. The recording system is managed by TINE SA, the largest dairy company in Norway. In 2018, approximately 95% of the Norwegian dairy herds were enrolled [25]. In NDHRS, individual cows are recorded as DD negative or DD positive by claw trimmers and veterinarians when trimming the claws. The inclusion criteria for herds in this study were at least one positive DD registration during the last trimming event, and that the professional trimmer responsible for registration in NDHRS was certified. Eligible herds were selected from three counties, Rogaland, Trøndelag, and Viken (Supplementary Fig. 1). These counties are animal-dense areas, with an expected large number of DD-positive dairy herds. Herds were selected based on in-herd-prevalence of DD and if possible, the herds with the highest prevalence were included in the study population. Fourteen herds trimmed by three certified trimmers in Rogaland, seven herds trimmed by two certified trimmers in Trøndelag, and one herd trimmed by one trimmer in Viken were included in the study.

### Study population

All sampling and clinical recordings in the present study were collected by an experienced veterinarian (the first author), and consisted of; M score of the DD lesions in the trimming chute after washing (The M-stage classification system defines DD lesions into M0, M1, M2, M3, M4, and M4.1 based upon macroscopic appearance [1, 26, 27]), biopsies from DD lesions, and swabs from the trimming equipment and chutes. Information about herd size, number of trimmed cows, proportion of DD-positive cows and M scores [1] is presented in Table 1.

**Table 1:** Herd size, cows trimmed, cows recorded with DD and different M-stage lesions [1]

	Number (n) (Median (min-max))	Proportion of trimmed cows (%)^1^ (Median (min-max))	Proportion of DD lesions (%)^2^ (Median (min-max))
Herd size^3^	59 (36–109)		
Cows trimmed	58 (33–78)		
DD + cows		39 (12–64)	
Recorded DD lesions according to M stages :			
M1			49 (14–82)
M2			2 (0–17)
M3			7 (0–33)
M4			10 (0–41)
M4.1			22 (0–50)

^1^DD+ cows/cows trimmed. ^2^Number of different M-stage lesions/total number of DD lesions. A cow can have more than one DD lesion. ^3^Herd size recorded in 2018, includes 22 Norwegian dairy herds

### Collection of swabs

The swabs were collected from February 20 to December 18, 2018. They were collected from nine locations on the trimming equipment and the chute (1–9). Detailed information about the sampling on each location is presented in Table 2. Sampling was performed at five different time points (A-E) on each farm; at arrival on the farm before trimming (A), after the trimming session (B), directly after washing with cold water (C), directly after washing with warm water without soap (D) and 15 min after disinfection (E). For each location, a sterile cotton swab was moistened with sterile 0.9% NaCl and swept over the surface for 10–20 s before being placed in a 1.5 ml Eppendorf tube filled with a nucleic acid stabilization solution (RNAlater®, Ambion, Austin TX) produced at the Technical University in Denmark (DTU) by co-author Tim Kåre Jensen. After collection, the swabs in RNA-later were stored at 4^o^ C until arrival at the Norwegian University of Life Sciences (NMBU) where they were frozen at -20^o^ C and later transported to the laboratory at DTU. At arrival, the tubes were immediately frozen at -20^o^ C. Due to staff problems related to Covid-19 at DTU, all frozen tubes were forwarded to the Swedish University of Agricultural Sciences (SLU) in November 2020. They were stored at -20^o^ C until DNA extraction and qPCR analysis for *Treponema* spp. and for *T. phagedenis, T. pedis,* and *T. medium /vincentii* from swabs collected at time point B, were performed.

**Table 2:** Locations for collection of swabs and description of the sampling area for each location

Location	Detailed description of sampling area^1^
1. Grinder – disc/cutting blades	Metal-backed grinding disks: from the whole surface of the disc Metal cutting disc: from the blades
2. Grinder – shield	From the inside of the shield
3. Grinder – handle	From the attachment of the handle to the grinder
4. Gloves	From the thumb and index finger of the right glove if right-handed, left glove if left-handed
5. Hoof knife	From the bended apex of the hoof knife
6. Hoof tester	From the rough area of the hoof tester´s jaw
7. Chute – footrest	From the whole surface, including an extra round over the two bolts attaching the footrest to the chute
8. Chute – cuff	From the area in contact with the limb (one cuff per chute)
9. Chute – belly belt^2,3^	From the middle part of the posterior edge of the rear belly belt

^1^ Photos of the sampling areas/locations are shown in Supplementary Fig. 2. ^2^ Not for the 8 first-visited herds (nr 2, 6, 11, 15, 16, 20, 21, and 22). ^3^ In eleven herds, the trimmers used chutes with belly belts made of textiles, and in three herds they were made of rubber

### Washing and disinfection of the trimming equipment and chutes

Information about the trimmers´ washing- and disinfection routines for cold water (C), warm water without soap (D), and disinfection (E) is presented in Table 3. Trimmer 1 trimmed eight herds, trimmer 2 and 3 five herds each, trimmer 4 two herds, and trimmer 5 and 6 trimmed one herd each.

**Table 3:** The six trimmers’ routines for washing and disinfection

Cold water routines (C)					
Trimmer	Grinder (1–3)	Gloves (4)	Hoof knife (5)/hoof tester (6)	Chute (7–9)	HPW temp. (^o^C)
1	HPW^1^	New^2^	HPW^3,4^	HPW^5^	< 10
2	Wash with brush and tap water	New	HPW	HPW	< 10
3	Disc/blades in water for 24 h, then manual wash	In washing machine	Hoof knife - manual wash at home. Hoof tester – with HPW when hanging on the chute	HPW	< 10
4	-	New	-	-^6^	< 10
4	HPW	New	HPW	HPW^7^	< 10
5	HPW	New	HPW	HPW	< 10
6	HPW	In washing machine	HPW	HPW	< 10
**Warm water routines without soap (D)**					
Trimmer	Grinder (1–3)	Gloves (4)	Hoof knife (5)/hoof tester (6)	Chute (7–9)	HPW temp.(^o^C)
1	HPW	-	HPW	HPW^8^	40
2	Wash with brush and tap water/ compressed air.	-	HPW	HPW	90
3	Manual wash	In washing machine at 60^o^ C	Hoof knife - manual wash at home/ Hoof tester - high pressure washer, hanging on the chute	HPW	80
4	HPW	-	HPW	HPW^7^	90
5	.	-	-	-^9^	90
6	HPW	In washing machine at 60^o^ C	HPW	HPW	50–60
**Disinfection routines (E)**					
Trimmer	Grinder (1–3)	Gloves (4)	Hoof knife (5)/hoof tester (6)	Chute (7–9)	Disinfection solution
1	Spray^10^	-	Spray^10^	Spray^10^	Vircon™ S 1%
2	Oven^11^	-	Oven^11^	Spray	Vircon™ S 1%
3	Spray before use	-	Spray before use	Spray	Salar disinfection 0.32% (TESS)
4	Spray^10^	-	Spray^10^	Spray and bucket^10^	Vircon™ S 1%
4	-	-	-	-^12^	Vircon™ S 1%
5	-	-	-	-^12^	Vircon™ S 1%
6	Spray	-	Spray	Spray	Vircon™ S 1%

^1^HPW = High pressure washer. ^2^New = Brand new gloves at each farm. ^3^Trimmer did not bring the hoof knife to herds 5, 9 and 21. ^4^Trimmer did not use a hoof tester. ^5^First wash with cold water (chute/grinder/hoof knife/hoof tester) was performed inside the barn. ^6^Wash with cold water not possible due to extremely cold weather. ^7^Chute and all trimming equipment were washed inside the trimmer’s truck. ^8^Chute was washed with warm water hanging from crane on the truck. Grinder/hoof knife/hoof tester were washed with warm water inside the barn. ^9^Due to lack of time, sampling was not performed after washing with warm water. ^10^Disinfection was performed on/inside truck. ^11^In oven at 90^o^C for 40 min. ^12^Due to lack of time, sampling was not performed after disinfection

**Fig. 1:**
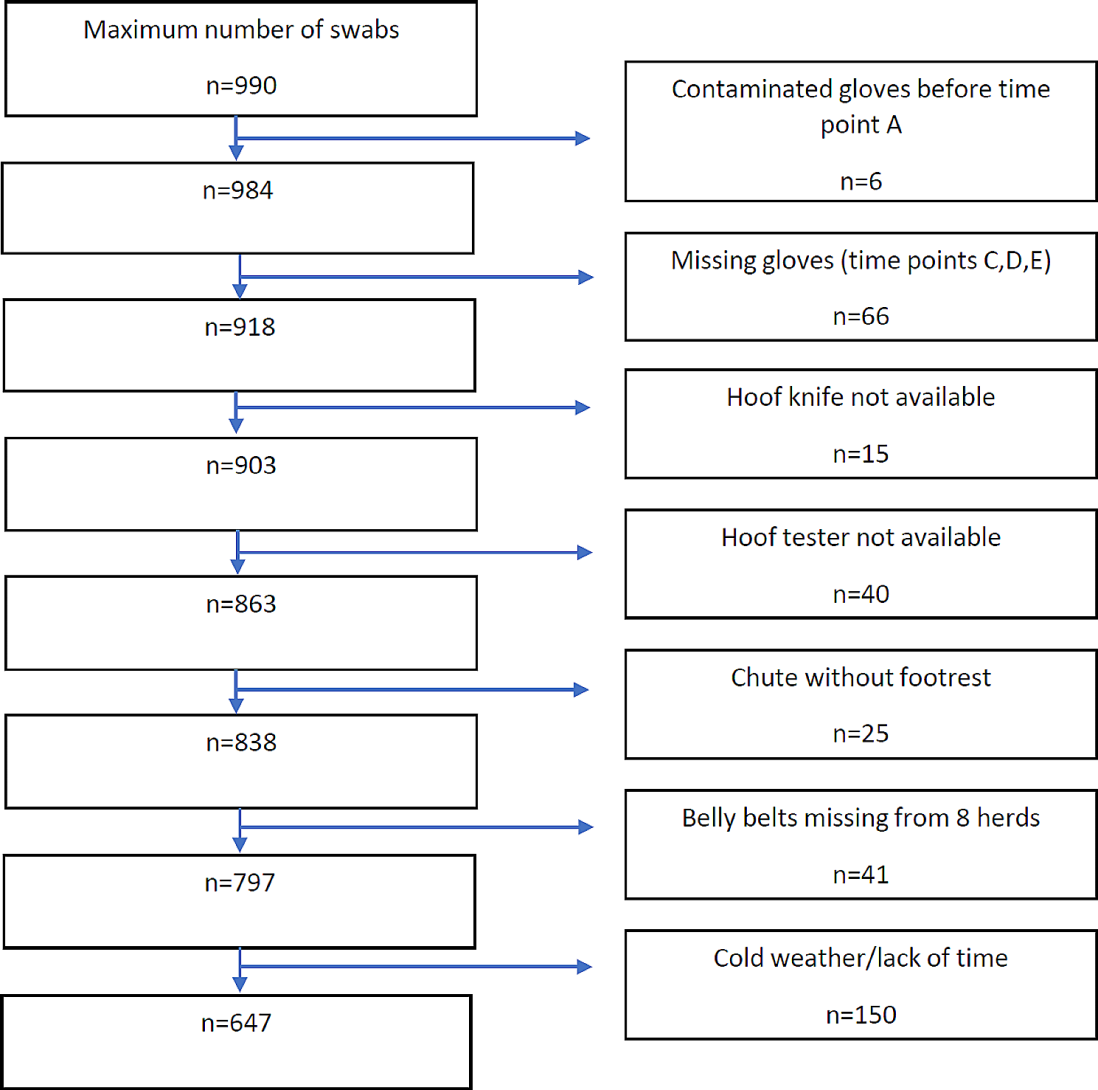
Flow chart of eligible swabs with reasons for exclusions

Washing with cold water (C), warm water without soap (D) and disinfection (E) on location 1–9, temperature of cold and warm water in degrees Celsius, and disinfection solution. In total, 647 swabs from 22 herds were collected from nine locations on the trimming equipment and chutes at five different time points. Altogether 343 swabs were missing for different reasons. Details are given in Fig. 1.

### DNA extraction and quantitative PCR analysis for *Treponema* spp. from swabs

Bacterial DNA was extracted from 647 swab samples using the EZ1 DNA Tissue Kit and the EZ1 DNA Bacteria Card in combination with the EZ1 Advanced XL instrument (Qiagen, Hilden, Germany). All swabs were pre-treated according to the manufacturer’s instructions for purification of bacterial DNA from primary samples (secretion swabs). In short, each swab was submerged in 290 µl G2 Buffer prior to the addition of 10 µl proteinase K (600 mAU/ml) and incubation at 56 °C and 600 rpm for 15 min. The swab was then removed and 200 µl sample was transferred to a 2.0 ml sample tube. Two micrograms carrier RNA (Qiagen) diluted in DNase- and RNase free water (Sigma–Aldrich, St Louis, MO, USA) was added to the sample tube before it was loaded into the EZ1 Advanced XL instrument (Qiagen). The elution volume used was 100 µl. Three negative controls were included which were prepared in the same way as the samples but without the swab.

The extracted DNA was used for analysis of *Treponema* spp. by the qPCR of Strub et al. 2007 with some modifications [28]. The TaqMan Fast Advanced Master Mix (Thermo Fisher Scientific Inc., Waltham, MA, USA) was used instead of TaqMan Universal PCR Master Mix (Thermo Fisher Scientific Inc.) and the total final volume was decreased from 25 µl to 15 µl. In addition, bovine serum albumin (BSA) (Sigma–Aldrich) was included at a final concentration of 0.1 mg/ml and the TaqMan Exogenous Internal Positive Control Reagents (Thermo Fisher Scientific Inc.) at 1 x concentration. PCR amplification was carried out in a CFX Opus 96 Real-Time PCR Instrument (Bio-Rad Laboratories Inc., Hercules, CA USA) using the following program: 10 min at 95 °C, followed by 45 cycles at 95 °C for 30 s and 60 °C for 1 min. The PCR run was analysed with the CFX Maestro Software version 2.0 (Bio-Rad Laboratories Inc.) with default settings. A total of 7 standards were prepared by 10-fold dilution series of *Treponema* pedis DSM18691 DNA (The Leibniz Institute DSMZ-German Collection of Microorganisms and Cell Cultures GmbH, Braunschweig, Germany) in DNase- and RNase free water (Sigma–Aldrich). The DNA concentration was determined using the Qubit ds DNA High Sensitivity Assay Kit (Invitrogen, Carlsbad, CA, USA) on the Qubit® 2.0 Fluorometer (Invitrogen). The standards were run in triplicates in each PCR run and a negative control consisting of DNase- and RNase free water (Sigma–Aldrich) was included in each run. Samples with probe-specific fluorescent signals within the highest and lowest dilutions of the standard curve were quantified. One copy number equals the DNA from one treponeme bacteria. The used detection limit for positive samples was copy number ≥ 3. No extrapolation of the standard curve was made, hence samples outside the standard curve were considered as negative for *Treponema* spp.

### Quantitative PCR analysis for detection of *T. phagedenis, T. pedis,* and *T. medium/vincentii* from swabs collected at time point B

All B samples taken directly after trimming but before washing and disinfection (*n* = 172) were analysed by a multiplex qPCR targeting *T. phagedenis, T. pedis,* and *T. medium/vincentii*, respectively [29]. The used detection limit for positive samples was copy number ≥ 10.

### Collection of biopsies

Altogether 109 biopsies were collected from cows with DD lesions in 21 of the herds included in the study population. The biopsies were collected with the animals restrained in the chute, during the same claw trimming events as the collection of the swabs. All biopsies were taken from one of the hind feet, after thorough cleaning of the area with water and a surgical scrub. Articare medical cold spray was sprayed onto the skin, for 10 s at 15 cm, immediately before biopsy puncture to reduce the pain. A 6 mm punch biopsy was stamped at the transition between the lesions and surrounding healthy skin. The biopsies were divided by a scalpel blade on a clean tray, into two equally sized parts, one half preserved in formalin and one half in RNA-later until analysis. All biopsies preserved in formalin were analysed by FISH at DTU in Denmark.

### Fluorescent in situ hybridization for detection of *Treponema* spp. in biopsies

For FISH analysis, the paraffin wax embedded biopsies sections of 4 μm were cut and mounted on SuperFrost + slides (Menzel-Gläser, Braunschweig, Germany), seventeen sections of each biopsy. The oligonucleotide probes used included probes specific for domain Bacterium, genus *Treponema, T. phagedenis, T. pedis, T. medium/vincentii,* and *T. refringens* as well as additional 12 phylotype specific oligonucleotide probes (from Nielsen et al. (2016)).

All biopsies were hybridized with the genus Treponema oligonucleotide probe as well as the 16 different Treponema specific oligonucleotide probes, each in combination with the domain Bacterium oligonucleotide probe. The slides were mounted in a Sequenza slide rack (Thermo Shandon, Cheshire, United Kingdom) and kept for 14 h in 45°C with 100µl of hybridization buffer (10 µl of 1 M Tris [pH 7.2], 18 µl of 5 M NaCl, 1 µl of 10% sodium dodecyl sulfate, 71 µl of H_2_O) with a concentration of 5 ng/µl of each applied oligonucleotide probe. The probe for domain Bacterium was 5’ labelled with fluorescein isothiocyanate (FITC) and all other bacteria oligonucleotide probes were 5’ labelled with the isothiocyanate derivative Cy3 (Eurofins MWG Operon, Ebersberg, Germany). The sections were then washed three times in pre-warmed (45 °C) hybridization buffer between 5 min interval and subsequently washed three times in pre-warmed (45 °C) washing buffer solution (10 ml of 1 M Tris [pH 7.2], 18 ml of 5 M NaCl, 72 ml of H_2_O) with the identical time interval. The sections were rinsed in water, air dried, and mounted in Vectashield (Vector Laboratories Inc., Burlingame, CA) for epifluorescence microscopy. An Axioimager M1 epifluorescence microscope equipped for epifluorescence with a 100-W HBO lamp and filter sets 43 and 38 was used to visualize Cy3 and FITC, respectively. Images were obtained using an AxioCam MRm version 3 FireWiremonocrome camera and AxioVision software, version 4.5 (Carl Zeiss, Oberkochen, Germany).

All biopsy specimens were originally scored from 0 to 3 according to the total bacterial invasion, and the prevalence of *Treponema* spp. according to Nielsen et al. (2016) [8] and Rasmussen et al. (2012) [5], however for this study the presence of *Treponema* spp. was only noted as +/-.

### Descriptive statistics

The outcome (copy number) was not normally distributed; hence confidence interval (CI) and mean could not be used. All data presented in figures, therefore includes median, 25^th^ and 75^th^ percentile, min and max values and outliers. Due to very high values for occasion B, we used logarithm of copy number (log_copynr) when presenting the data in boxplots, making visualization of the data clearer. However, log 0 is undefined and not a real number, meaning that log 0 produces missing values. To avoid losing negative samples when transforming our copy numbers to log_copynr we added the value of 1 to all copy numbers (log_copynr = log_(copynr + 1)).

## Results

### Quantitative PCR analysis for detection of *Treponema spp.* in the swabs

Table 4 presents the distribution of positive samples (number and proportion) for *Treponema* spp. from locations 1 to 9 on the trimming equipment and chutes at time points A to E. The three locations on the chute (footrest, cuff, and belly belt) had the highest proportion of positive samples 15 min after disinfection (0.25, 0.33 and 0.67) compared to the other trimming equipment.

**Table 4:** Number of swabs and positive proportion after quantitative PCR analysis for *Treponema* spp.

	Sampling time points of swabs											
	At arrival (A)		After trimming (B)		After cold water (C)		After warm water without soap (D)		After disinfection (E)		In total	
Sampling location	n	+/n	n	+/n	n	+/n	n	+/n	n	+/n	n	
Grinder-disc/blades	22	0.50	22	1.00	15	0.73	14	0.43	11	0.18	84	
Grinder-shield	22	0.55	22	1.00	16	0.81	16	0.56	10	0.20	86	
Grinder-handle	22	0.59	22	1.00	16	0.81	14	0.57	11	0.09	85	
Glove	14	0.29	21	1.00	-	-	-	-	-	-	35	
Hoof knife	19	0.32	19	1.00	10	0.60	12	0.58	9	0.00	69	
Hoof tester	14	0.36	14	1.00	10	0.60	13	0.23	6	0.17	57	
Chute-footrest	16	0.44	16	1.00	15	0.80	14	0.57	12	0.25	73	
Chute-cuff	22	0.50	22	1.00	18	0.89	20	0.65	15	0.33	97	
Chute-belly belt	13	0.77	14	1.00	12	1.00	13	0.92	9	0.67	61	
Total	164	0.48	172	1.00	112	0.79	116	0.57	83	0.24	647	

**Fig. 2:**
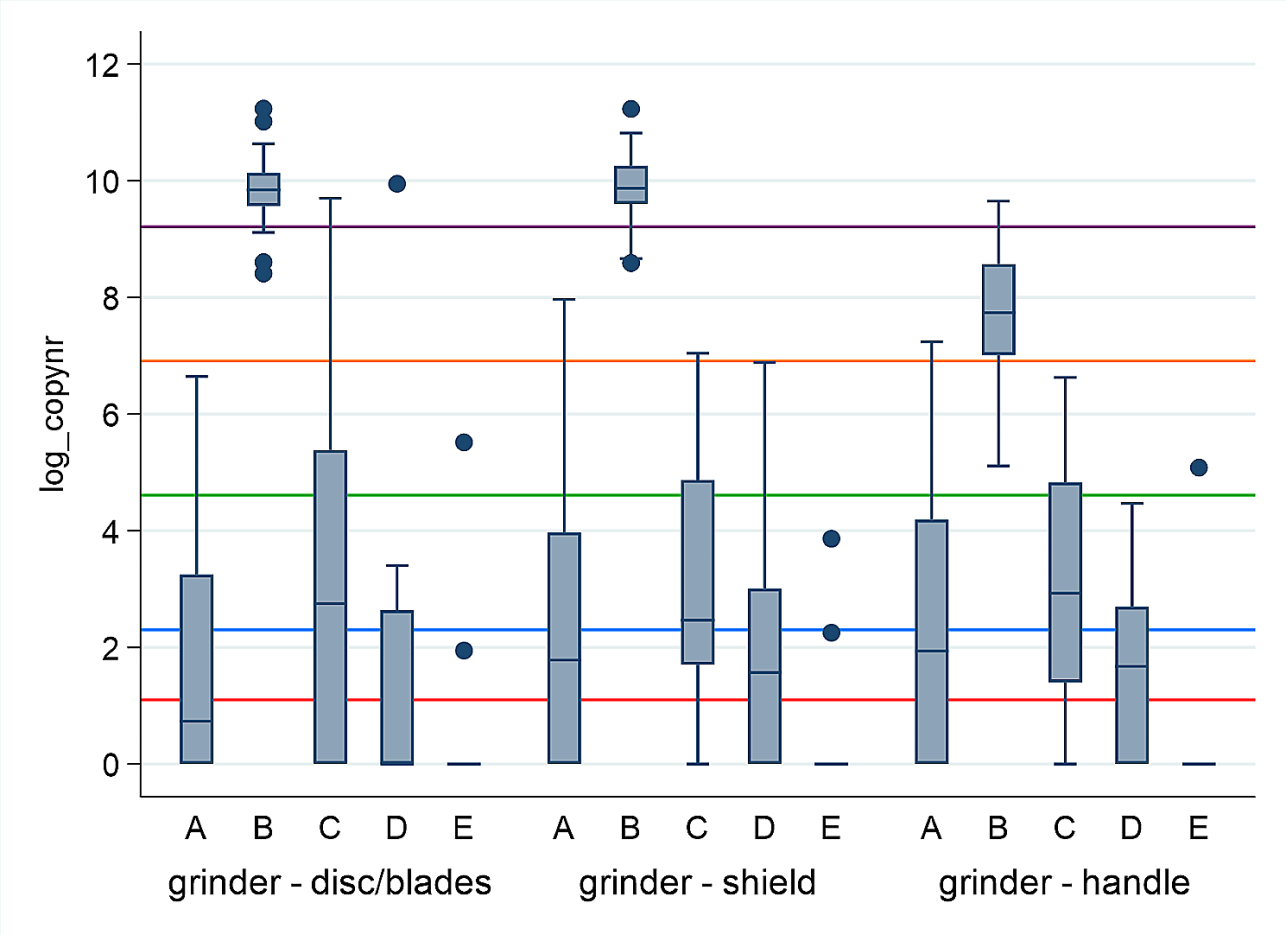
Log_copy numbers (median, 25^th^ and 75^th^ percentile, min, max and outliers) after qPCR for *Treponema* spp. from 3 locations on the grinder (disc/cutting blades, shield, and handle), collected at five different time points (**A**-**E**), in 22 Norwegian dairy herds. A = At arrival on farm before trimming, B = After ended trimming session, C = After washing with cold water, D = After washing with warm water without soap, E = 15 min after disinfection. The red line equals copy number = 3 (detection limit). The blue line = copy number 10. The green line = copy number 100. The orange line = copy number 1000. The purple line = copy number 10 000

**Fig. 3:**
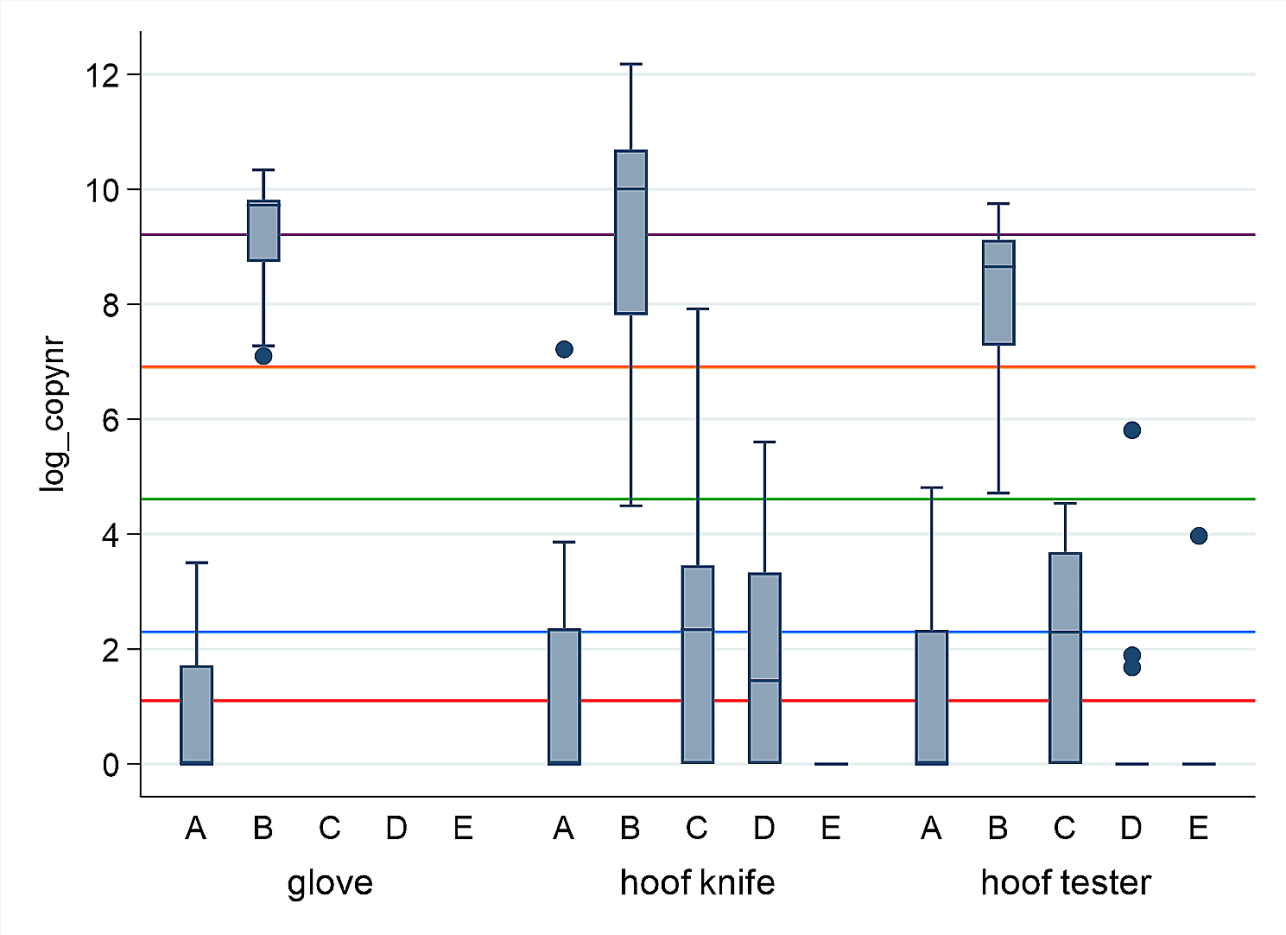
Log_copy numbers (median, 25^th^ and 75^th^ percentile, min, max and outliers) after qPCR for *Treponem*a spp. from glove, hoof knife, and hoof tester at five different time points (**A**-**E**), in 22 Norwegian dairy herds. A = At arrival on farm before trimming, B = After ended trimming session, C = After washing with cold water, D = After washing with warm water without soap, E = 15 min after disinfection. The red line equals copy number = 3 (detection limit). The blue line = copy number 10. The green line = copy number 100. The orange line = copy number 1000. The purple line = copy number 10 000

**Fig. 4:**
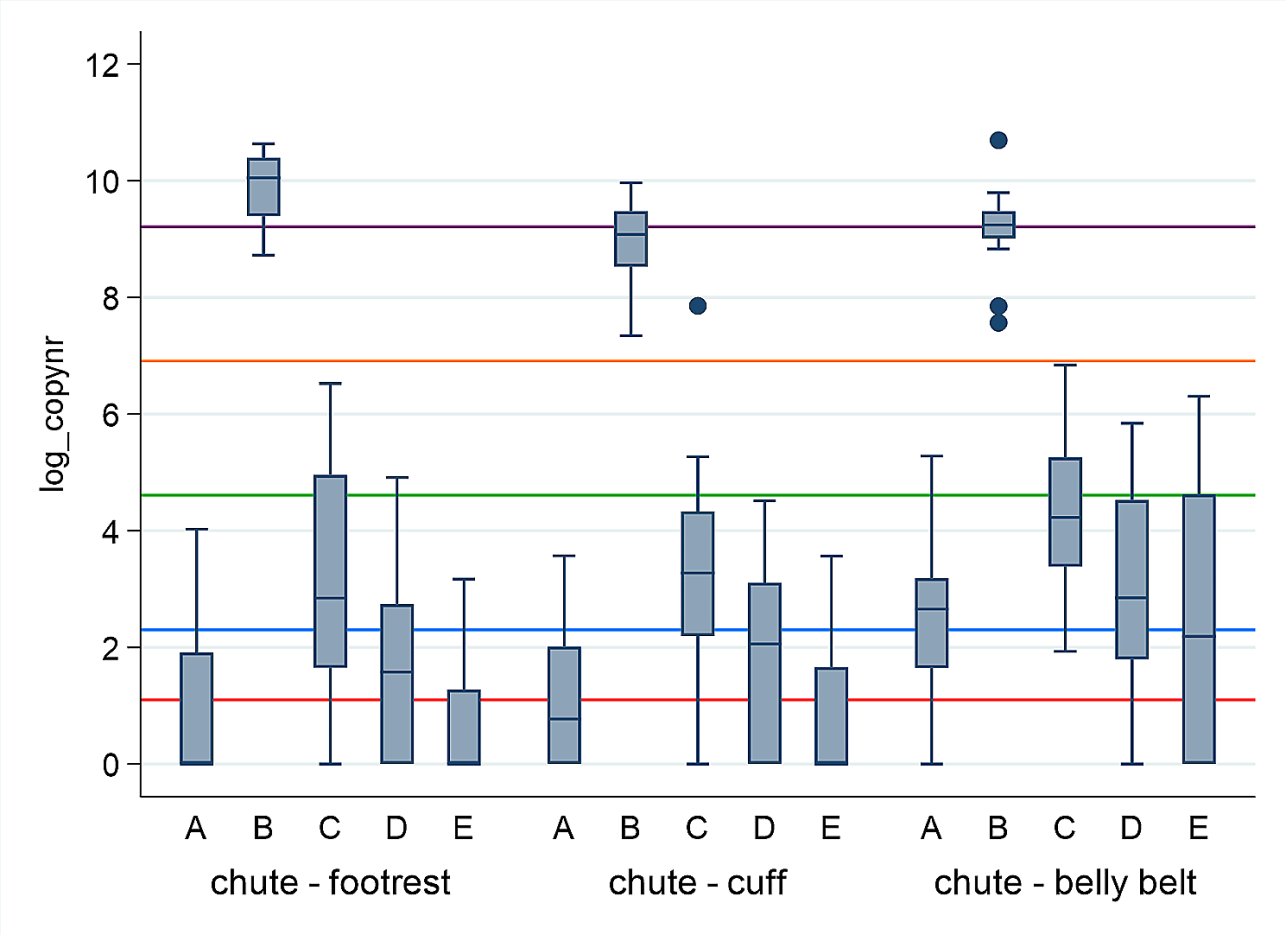
Log_copy numbers (median, 25^th^ and 75^th^ percentile, min, max and outliers) after qPCR for *Treponema* spp. from 3 locations on the chute (footrest, cuff, and belly belt), collected at five different time points (**A**-**E**), in 22 Norwegian dairy herds. A = At arrival on farm before trimming, B = After ended trimming session, C = After washing with cold water, D = After washing with warm water without soap, E = 15 min after disinfection. The red line equals copy number = 3 (detection limit). The blue line = copy number 10. The green line = copy number 100. The orange line = copy number 1000. The purple line = copy number 10 000

**Fig. 5:**
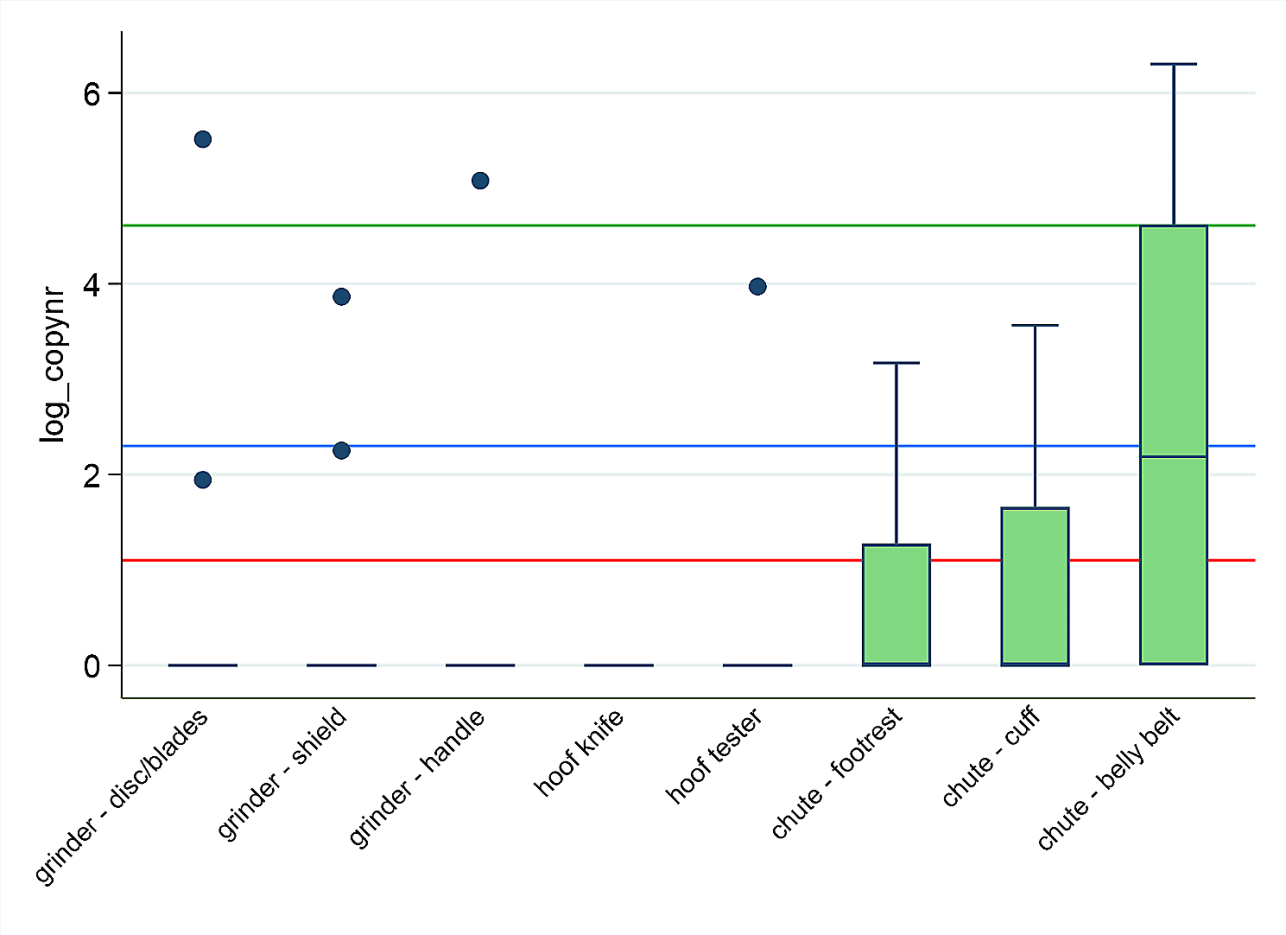
Log_copy number (median, 25^th^ and 75^th^ percentile, min, max) after qPCR for *Treponema* spp. from 3 locations on the grinder, the hoof knife, the hoof tester, and 3 locations on the chute (altogether 8 locations) 15 min after disinfection in 22 Norwegian dairy herds. The red line equals copy number = 3 (detection limit). The blue line = copy number 10. The green line = copy number 100

The log_copy numbers after qPCR for *Treponema* spp. of 647 swabs collected from nine specific locations, at five different time points (A-E) are presented in Figs. 2, 3, 4 and 5. Large differences in minimum and maximum values for the same locations verify big difference in *Treponema* spp. load between the herds. The highest copy numbers for all nine locations are seen immediately after trimming (B). There is a decline in copy numbers when comparing swabs collected after washing with cold water (C versus B) and washing with warm water without soap (D versus C) for most locations. After disinfection (E), few samples were positive for *Treponema* spp. (copy number ≥ 3), and no treponemes (viable or not) were detected on the hoof knife.

Figure 2 shows that the disc/cutting blades (median = 18858.6, max = 75730.5) and the shield (median = 19348.2, max = 75398.3) on the grinder had higher copy numbers compared to the handle (median = 2290.0, max = 15490.6) immediately after trimming (**B**). The figure also shows that swabs from all locations on the grinder had higher copy numbers at arrival on the farm (**A**) compared to 15 min after disinfection (**E**) 

Figure 3 shows that the hoof knife had higher copy numbers (median = 22169.2, max = 194584.4) versus the hoof tester (median = 5890.0, max = 17109.3) immediately after trimming (**B**). Five of the six swabs from the hoof tester were negative 15 min after disinfection (**E**) (max = 52.0). No *Treponema* spp. was detected in the nine samples from hoof knives after disinfection (**E**). The hoof knife and hoof tester also had higher copy numbers at time point A compared to time point E.

Figure 4 shows that the footrest had the highest copy numbers immediately after trimming (B) (median = 23162.4, max = 41502.0) versus both the cuff (median = 8769.8, max = 21249.5) and the belly belt (median = 10326.6, max = 17952.8 (outlier = 44069.1)), but the lowest 15 min after disinfection (median = 0, max = 22.8). The belly belt had the highest copy numbers after disinfection (E) (median = 7.9, max = 545.1)

The log_copy numbers after qPCR for *Treponema* spp. from eight locations, collected 15 min after disinfection (E) are presented in Fig. 5. All samples from the hoof knives were negative after disinfection. The belly belt had highest number of positive samples (6/9) and highest copy numbers (median = 7.9, max = 545.1) 15 min after disinfection. The only positive belly belt made of rubber had a very low positive copy number (3.5) compared to the five positive samples from belly belts made of textiles (7.9, 62.6, 101, 118, and 545).

### Quantitative PCR analyses for detection of *T. phagedenis, T. pedis,* and *T. medium/vincentii* from swabs collected at time point B

*Treponema phagedenis* was detected in swabs from all herds except from 1 to 19. In the twenty positive herds, *T. phagedenis* was detected in a minimum of two locations on the trimming equipment and chutes with a median of four and a maximum of seven. *Treponema pedis* and *T. medium/vincentii* were not detected. The results of the qPCR analyses of *T. phagedenis* in swabs collected from trimming equipment and chutes immediately after trimming (time point B) in 22 Norwegian dairy herds are presented in Table 5.

### FISH analyses for detection of *Treponema spp. in biopsies from DD lesions*

Results of the *Treponema* phylotypes identified by FISH analyses in collected biopsies from different cows with DD lesions at the same trimming event are presented in Table 5. In total, 16 phylotypes/species were examined by FISH, but only *T. phagedenis, T. pedis, and T. refringens*, were identified. *Treponema phagedenis* was identified in biopsies from all herds, except from 1 and 19. However, all biopsies from herd 1 and 19 were positive for *Treponema* organisms.

**Table 5:** Biopsies identified with *Treponema* phylotypes^1^ by FISH^2^, and swabs analyzed with qPCR for *T. phagedenis*^1^

Biopsies analysed by FISH (n)							Swabs analysed by qPCR for *T. phagedenis* at time point B	
Herd	n^3^	*T. phagedenis*	*T. pedis*	*T. medium/ vincentii*	*T. refringens* ^*3*^	*Genus Treponema*	Locations swabbed^5^ n	*+* n
1	6	0	0	0	0	6	9	0
2	5	5	0	0	1	5	7	5
3	5	5	4	0	0	5	9	3
4	5	3	0	0	0	5	7	4
5	5	3	1	0	3	4	7	5
6	6	6	3	0	0	6	7	6
7	5	4	2	0	4	4	8	3
8	4	4	4	0	4	4	8	7
9	5	5	1	0	4	5	7	6
10^4^	-	-	-	-	-	-	9	5
11	6	6	0	0	0	6	7	4
12	5	5	3	0	0	5	9	3
13	4	2	0	0	0	4	9	3
14	5	5	2	0	0	5	8	2
15	5	4	1	0	0	5	7	4
16	6	3	2	0	0	5	7	3
17	6	6	0	0	0	6	9	4
18	6	4	3	0	0	6	9	6
19	5	0	0	0	0	5	9	0
20	5	5	2	0	0	5	7	4
21	4	2	1	0	0	3	6	3
22	6	6	0	0	0	6	7	3

Collected biopsies from 109 DD cows identified with *Treponema* phylotypes (n) by FISH analyses and results of qPCR analyses for *T. phagedenis* (+) in 172 swabs collected from trimming equipmentand chutes immediately after trimming in 22 Norwegian dairy herds (n).

^1^All biopsies were analyzed for altogether 16 phylotypes, but only *T. phagedenis*, *T. pedis,* and *T. refringens* were identified. ^2^One DD lesion can be positive for multiple *Treponema* phylotypes at the same time. ^3^*T. refringens*/PT3 = GenBank sequence database with accession number AM942447 [30]. ^4^No biopsies were collected from herd 10. ^5^All possible 9 locations: The grinder (disc/cutting blades, shield, handle), glove, hoof knife, hoof tester, chute (footrest, cuff, belly belt). *Treponema phagedenis* on the different swabbed areas is shown in Supplementary Table 1.

## Discussion

We found large differences in copy number of *Treponema* spp. depending on herd, location on the trimming equipment and chute, and time points for collection of swabs. There was a decrease in *Treponema* spp. load from samples collected after washing and disinfection versus those collected immediately after trimming. The disc/blades on the grinder and the belly belt on the chute had the highest copy numbers after disinfection. The belly belt and the cuff had the highest number of positive samples after washing and disinfection (6/9 and 5/15). The DD-associated phylotype *T. phagedenis* was detected in swabs from two or more locations from all herds except from herd number 1 and 19.

The large differences in copy numbers for *Treponema* spp. immediately after trimming (B) could partly be explained by different DD prevalence in the herds (Table 1). The high proportion of M1 stages compared to other studies may be explained by how the DD lesions were diagnosed and recorded. Depending on how clean the feet are and how thoroughly the diagnostics are performed, visual inspection may identify the presence of DD lesions with varying results. In a Finnish study the feet were examined on standing cows with a mirror without washing the feet before scoring [31]. In a Canadian study, Ferraro et al. (2020) used a borescope in the milking parlour without cleaning the feet [32]. In the present study the cows’ feet were examined in the trimming chute after fixation, washing and with good lighting. A large proportion of the registered M1 lesions in the present study were located in the interdigital cleft, which would have been difficult to detect and record with a mirror or a borescope. Another explanation for the high M1 proportion and a relatively low M4 proportion may be that DD is a relatively new disease in Norway which has appeared during the last 10 years [33]. The within herd prevalence of DD is still low in Norwegian dairy herds, and in this situation a high proportion of M1 lesions is probably as expected. Another explanation could be related to breed. The majority of cows in Norway are Norwegian Red. It has been discussed that Norwegian Red could be more resilient to treponemes and the development of DD lesions. However, there are no studies to support this statement.

The differences in within herd prevalence of DD could be explained by different biosecurity routines and by different hygiene and cleaning routines in the individual barns. Poor hygiene in the cubicles and alleys with dirty and moist environment results in maceration of the skin with reduced resistance to bacteria, making the cows more susceptible to DD lesions, and secondarily increasing the load of DD-associated *Treponema* spp. in the environment [34–37]. Poor hygiene also increases the load of treponemes originating from faeces [38].

Another important variable having a huge impact on the copy numbers for time points A, C, D and E is the trimmer responsible for the washing and disinfection of the trimming equipment and chute. The six trimmers had different routines (Table 3), which directly influence the results. Unexpected random situations occasionally happen on farms, which may disturb the washing and disinfection procedures, and cause outliers with high copy numbers of *Treponema* spp. However, statistical analysis of the impact of the trimmer was not possible due to the study population, e.g., one trimmer was responsible for the trimming in eight herds while two other trimmers only were responsible for one herd each. In addition, it can be difficult to distinguish between the effect from the trimmer versus the herd.

Decreased *Treponema* spp. load after washing with cold water (C) with further decrease after washing with warm water (D) agrees with Gillespie et al. (2020) who found that water was effective to remove viable treponemes from hoof knives [39]. However, Angell et al. (2017) showed that washing gloves used at trimming feet in sheep flocks with contagious ovine digital dermatitis (CODD) with warm and cold water, was ineffective in preventing detection of DD treponemes by PCR and culture [21]. Their study and a further decrease in copy number after disinfection (E) for most locations in the present study, strongly indicates that disinfection should be recommended after routine washing to minimize the risk of live treponemes on the trimming equipment and chute. The disinfectant Virkon™ S was chosen and used by most claw trimmers in the present study, probably because it is easily available with well-documented effect even though no studies have reported this biocide’s efficacy against microorganisms present in biofilms [40]. Virkon™ S is characterized by being non-corrosive to stainless steel, having a low ecotoxicity / high biodegradability, and low toxicity [41]. According to the safety sheet emitted by Lanxess it can cause severe eye damage, skin and respiratory irritation [42].

We cannot exclude that the decrease in *Treponema* spp. load in the present study might be influenced by repeated high-pressure washer, and not the water temperature. One could argue that the frequency of the high-pressure washer has the same effect regardless of temperature due to the pressure blowing the dirt away. On the other hand, cold water is known for removing biological material without denaturing proteins, and warm water is known for dissolving fat (preferable with soap), both important steps in removing biological material prior to disinfection [43, 44]. The necessity of removing manure before disinfection is supported by Hartshorn et al. (2013) who in their in vitro study found that the effectiveness of many different disinfectants used in foot baths, especially copper sulphate, was reduced when manure was present [23]. Results from the study by Geraldes et al. (2021) indicated that organic matter inactivated Virkon™ S and highlighted the importance of cleaning surfaces thoroughly before disinfecting with this biocide. Staton et al. (2020) recommend the use of soap in their disinfection protocol and washing with soap in step 2 would probably have been beneficial to remove manure, but was not included in the present study [24].

Big differences in load of *Treponema* spp. between the different trimming equipment and different locations on the chute after washing and disinfection may partly be explained by how rough/uneven and complex the fomites were. The hoof knife has a smooth surface, and consequently the removal of manure and other organic material is easy. High *Treponema* spp. load on the discs/blades of the grinder may be caused by the rough, uneven surfaces, which is experienced to be more difficult to clean. The high number of positive samples on the belly belt and cuff, and the high copy numbers of *Treponema* spp. may be explained by most of these fomites being woven textiles and difficult to wash and disinfect. Recently many claw trimmers have exchanged the belly belts made of textiles with rubber belts which anecdotally is easier to clean and disinfect.

Increased bacterial load at arrival on the farm (A) versus after disinfection (E) might be explained by the fact that the trimming equipment and chutes were not properly cleaned and disinfected after the previous trimming. Recontamination with faeces after disinfection could also be an explanation because many of the trimmers wash at farm (inside or outside the barn), and sometimes in wet and muddy conditions. The trimmers also might have improved their washing and disinfection procedures during the present study. Recontamination may also be an important factor for the extreme values (outliers) after washing with warm water and disinfection in the present study.

In the present study *T. phagedenis* was detected in swabs from all herds except two. No finding of *T. medium* agrees with the FISH analyses of the biopsies in Table 5. This table also shows that *T. pedis* was identified in few biopsies (29/109). *Treponema pedis* may have been present on some of the swabs, but below the detection limit (copy number ≥ 10). These findings agree with a Norwegian study of interdigital dermatitis, heel horn erosion, and digital dermatitis in 14 dairy herds where seven different phylotypes were detected by FISH analysis, however none were *T. medium* or *T. pedis* [45].

Detection of *T. phagedenis* in swabs from all herds except 1 and 19 agrees with the results from the FISH-analyses (Table 5) made on the biopsies collected from DD lesions of cows in the same herds. Most-recently performed 16 S sequencing on the second half of the same biopsies also identified *T. phagedenis* positive biopsies from all herds except 1 and 19, in these two herds *T. refringens* was identified (personal communication Bjørn Spilsberg). This strongly indicates that *Treponema* spp. from DD lesions were present on the trimming equipment and chutes even though, they probably also were contaminated with faeces which is proven to be a reservoir for *Treponema* spp.. Zinicola et al. (2015) [38] found DD-associated *Treponema* spp. nearly ubiquitously in rumen and fecal microbiota and suggested that the gastrointestinal tract is a reservoir of microbes in the DD pathogenesis. However, Klitgaard et al. (2017) [34] found in a study of slurry from dairy herds with and without DD-infected cows, that DD-associated *Treponema* spp. were only found in slurry from herds with reported DD. They suggested that slurry is not a primary reservoir of DD infection.

Sullivan et al. (2014) [17] found DNA from DD-associated treponemes on the blades of hoof knives after trimming DD-positive cattle and sheep. The results from the present study and Sullivan’s study implies that the trimming equipment and the chute might transfer DD-associated *Treponema* spp. to other cows within the same herd and may potentially be a passive vector of DD-associated treponemes to other and naïve herds if recommended washing and disinfection routines are not implemented.

A weakness of the present study is that no cultivation of bacteria was performed, and the PCR cannot distinguish between viable and non-viable treponemes. On the other hand, the agreement between the results from the qPCR analyses for *T. phagedenis*, *T*. *pedis*, *T*. *medium*/*vincentii* of the B-samples, and the results from the FISH-analyses of the collected biopsies from DD lesions in the same herds, indicates that some treponemes identified on the trimming equipment and chutes originated from DD lesions on the trimmed cows. It is therefore important with thorough washing routines to remove all biological material in which the treponemes can survive for longer periods. Gillespie et al. found that DD-associated treponemes may survive for up to 2 h under aerobic laboratory conditions [46]. Bell et al. found that DD treponemes were viable for seven days in sand bedding, six days in sawdust, and five days in recycled manure solid microcosms [47]. Field conditions where the bacteria can hide in faecal material or claw horn after trimming, may probably keep them viable substantially longer. In Norway, it is not unusual that professional trimmers visit more than one herd the same day, which makes recommended washing- and disinfecting routines important to prevent transmission of viable DD-associated *Treponema* spp. between herds, many of them naïve with no cows previously recorded with DD lesions. This study shows that the chute is especially difficult to clean and disinfect and probably imply an even greater risk regarding passive transmission of treponemes compared to the trimming equipment. The Norwegian guidelines regarding washing and disinfection of the trimming equipment and chutes include: a first wash with lukewarm water below 50^o^ C with high-water pressure and a brush to remove dirt. Then alkaline soap is applied with low to moderate pressure to work for at least 10 min. Finally, the soap is rinsed of. The equipment should dry before disinfection [48].

It is also a weakness that qPCR analyses for *T. phagedenis*, *T*. *pedis*, and *T*. *medium*/*vincentii* were only performed of the B-samples. All time point samples should preferably have been analysed for these DD-associated phylotypes, but restricted resources available made this impossible. We chose time point B which showed the situation immediately after trimming. It would have been very interesting to also analyse for these DD-associated phylotypes at time point E, not only for *Treponema* spp. However, there is no reason that these DD-associated phylotypes including *T. phagedenis* would respond differently to the disinfectants used by the certified trimmers.

A larger study population, including a control group for instance including the use of soap, may have been preferable. However, the swabs in the present study were collected at routine trimming performed by certified trimmers performing their own washing and disinfection routines. By doing so, the sampling procedure and results were representative for the trimmers’ routines. On the other hand, predetermined and equal washing and disinfection routines could have yielded more samples and made it easier to interpret the results.

Due to very cold weather during some periods of the fieldwork, all steps of the washing and disinfection routines were not possible to perform in all herds, making the number of swabs less than planned. The optimal period for collecting swabs was May to October, but this was not possible to arrange due to the trimmers´ fully booked schedules.

As far as we know no other studies have collected and analysed swabs from so many different tools and locations on the trimming equipment and chute.

## Conclusions

This study shows that DD-associated *Treponema* spp. (viable or not) were present on the trimming equipment and chutes after trimming cows in DD-positive herds. Washing and disinfection reduced the load of *Treponema* spp.. Large differences in *Treponema* spp. load between the different locations were documented. High copy numbers of *Treponema* spp. on the grinder, and on all three locations on the chute after disinfection, indicates that sufficient cleaning and disinfection of these locations is difficult to perform, and therefore have the potential for passive transfer of DD-associated treponemes. This knowledge is valuable to prevent further dissemination of the disease between herds.

### Electronic supplementary material

Below is the link to the electronic supplementary material.

Supplementary Material 1

Supplementary Material 2

Supplementary Material 3

## Data Availability

The datasets generated and analyzed during the current study are available in the repository named BioStudies. (www.ebi.ac.uk/biostudies). Identification number: S-BSST1066.

## Declarations

### Author information

LA graduated as a veterinarian in 2014 and is currently a Ph.D.-student in the project “Digital dermatitis – a contagious claw disease threatening animal welfare” at the Norwegian University of Life Sciences (NMBU). TF is professor in surgery, lameness, and claw diseases in farm animals at NMBU. IHH is a veterinary epidemiologist and associate professor at the Ambulatory Clinic and Herd Health Services at NMBU. ÅMS is a specialist veterinarian with PhD, in the Norwegian Cattle Health Services in Animalia. TKJ is a professor and veterinary pathologist at Department of Veterinary and Animal Sciences, University of Copenhagen. SF and AR are Researchers in Bacteriology at the Department of Biomedical Sciences and Veterinary Public Health, Swedish University of Agricultural Sciences (SLU).
