# Supplementary material for: Bovine Digital Dermatitis: Treponema spp. on trimming equipment and chutes – effect of washing and disinfection
Source: BMC Vet Res. 2024 Jun 18;20:261. doi: 10.1186/s12917-024-03941-z (PMC11184789; doi:10.1186/s12917-024-03941-z)
Supplement: Supplementary file 1 — Supplementary Material 1 [file 12917_2024_3941_MOESM1_ESM.pdf]

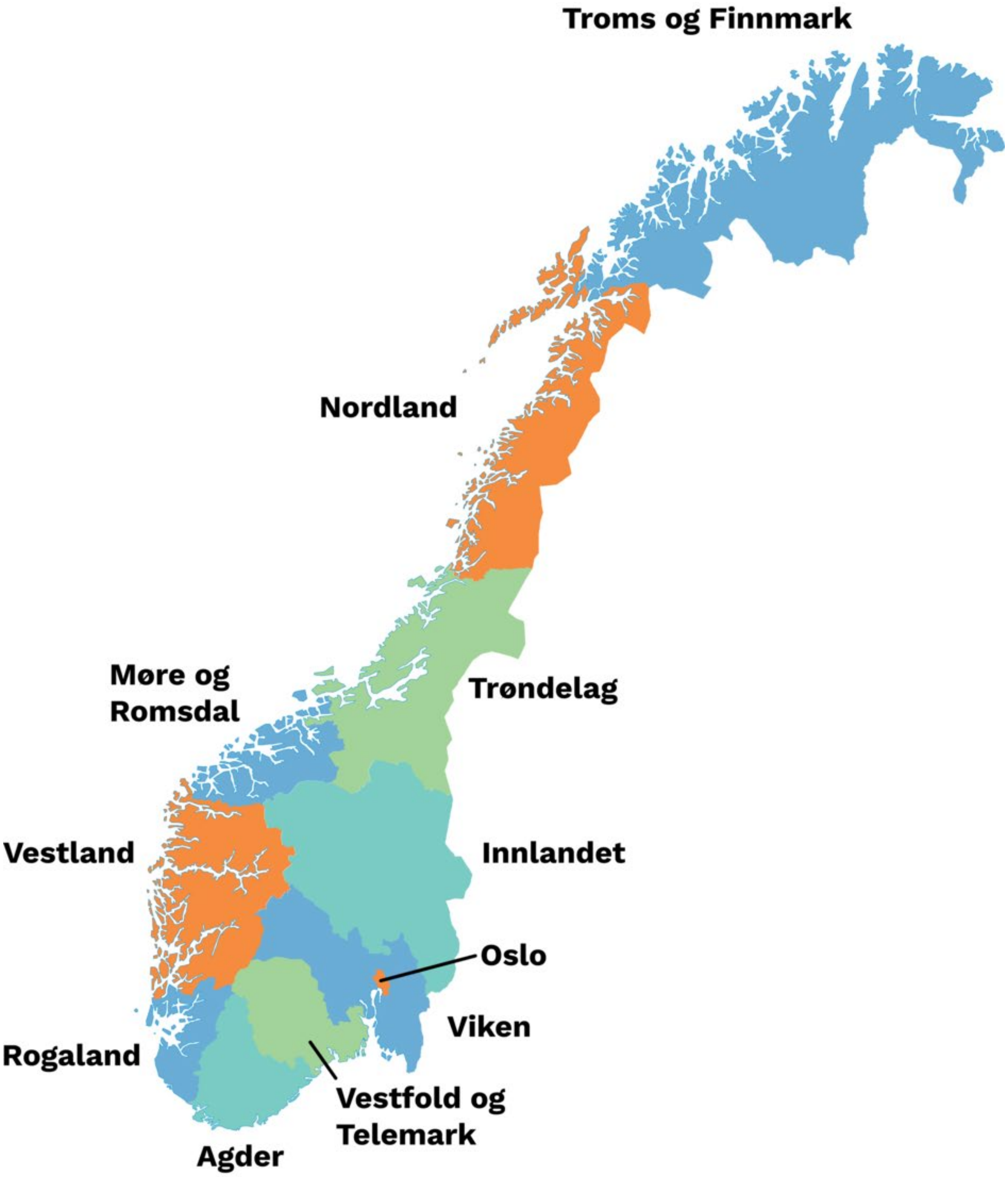

Supplementary Figure 1 The counties of Norway from 2020 to 2023

By Erik Bolstad/Store norske leksikon.
https://snl.no/fylke
