# Supplementary material for: Bovine Digital Dermatitis: Treponema spp. on trimming equipment and chutes – effect of washing and disinfection
Source: BMC Vet Res. 2024 Jun 18;20:261. doi: 10.1186/s12917-024-03941-z (PMC11184789; doi:10.1186/s12917-024-03941-z)
Supplement: Supplementary file 2 — Supplementary Material 2 [file 12917_2024_3941_MOESM2_ESM.pdf]

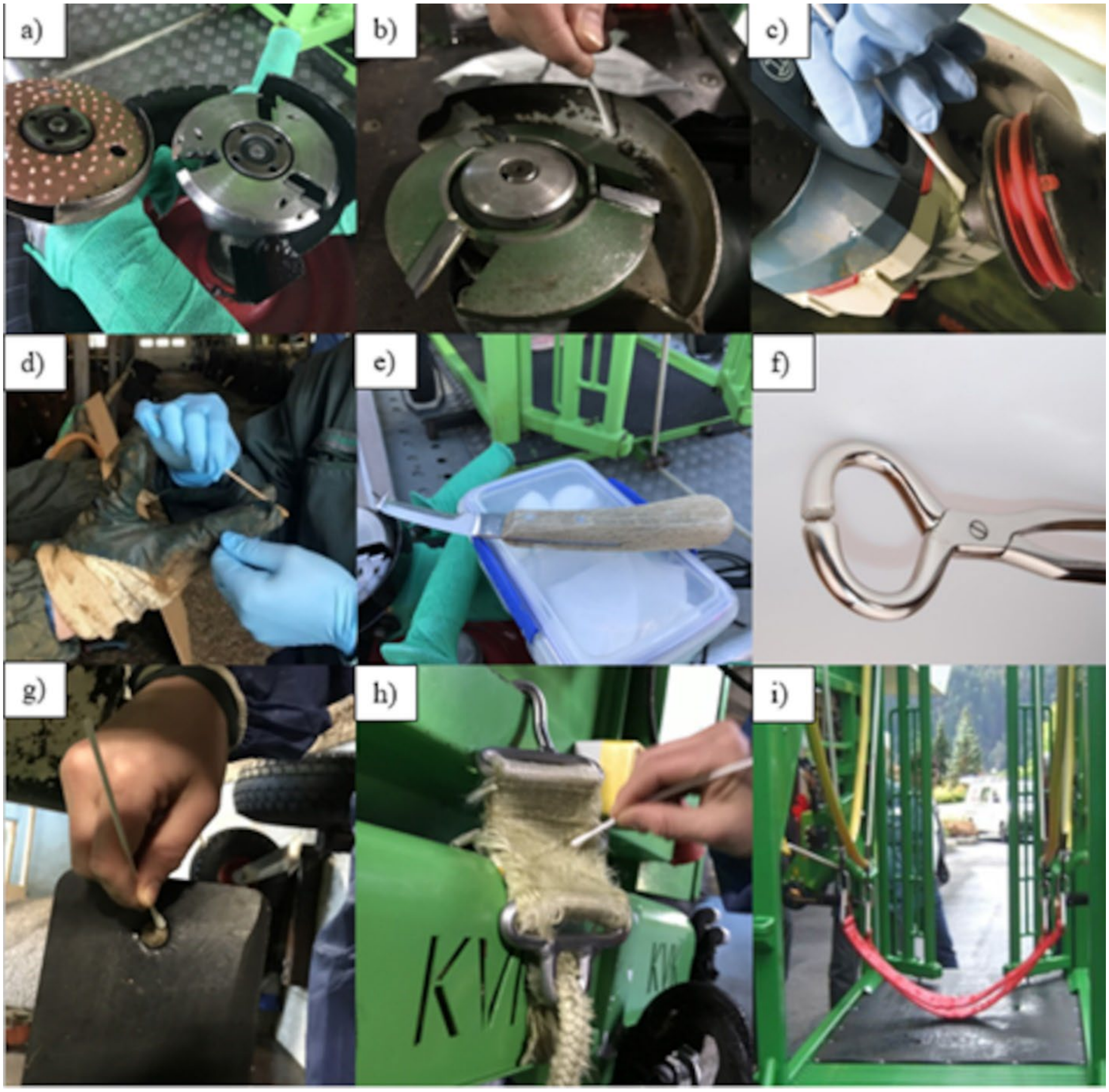

Supplementary Figure 2 The nine locations on the trimming equipment and chute where swabs were collected for qPCR analysis[1]

a) Grinder – disc/cutting blades b) Grinder – inside of the shield c) Grinder – attachment of the handle d) Gloves – thumb and index finger e) Hoof knife – bended apex of the blade f) Hoof tester – the rough area of the tester's jaw[2] g) Chute –footrest h) Chute – cuff i) Chute – belly belt

[1]qPCR-analysis for *Treponema* spp., including DD-associated treponemes. [2]Photo from https://www.heimer.no/hovslager/verktoy/annet-verktoy/praktiske-ting/visitertang-liten
