# Supplementary material for: Bovine Digital Dermatitis: Treponema spp. on trimming equipment and chutes – effect of washing and disinfection
Source: BMC Vet Res. 2024 Jun 18;20:261. doi: 10.1186/s12917-024-03941-z (PMC11184789; doi:10.1186/s12917-024-03941-z)
Supplement: Supplementary file 3 — Supplementary Material 3 [file 12917_2024_3941_MOESM3_ESM.pdf]

Supplementary Table 1 qPCR analysis of *T. phagedenis*[1] in swabs collected[2] from trimming equipment/chutes

Herd[3]	Location swabbed[4] (n)	*T. phagedenis* + (n)	*T. phagedenis* on the different locations								
			1	2	3	4	5	6	7	8	9
1	9	0	-	-	-	-	-	-	-	-	-
2	7	5	+	-	-	+	+	+	m[5]	+	m
3	9	3	-	+	-	+	-	-	+	-	-
4	7	4	+	+	+	m	+	-	m	-	-
5	7	5	+	-	-	+	m	m	+	+	+
6	7	6	+	+	+	+	+	m	-	+	m
7	8	3	-	-	-	+	+	-	m	+	-
8	8	7	+	+	+	+	+	-	m	+	+
9	7	6	+	+	-	+	m	m	+	+	+
10	9	5	-	+	-	+	+	-	+	-	+
11	7	4	+	-	-	+	+	+	m	-	m
12	9	3	+	-	-	+	+	-	-	-	-
13	9	3	-	+	+	+	-	-	-	-	-
14	8	2	-	-	-	-	-	m	+	-	+
15	7	4	+	+	-	+	-	m	+	-	m
16	7	3	-	-	+	+	+	m	-	-	m
17	9	4	+	+	-	+	-	-	-	-	+
18	9	6	+	+	-	+	+	-	+	-	+
19	9	0	-	-	-	-	-	-	-	-	-
20	7	4	+	-	-	+	+	+	m	-	m
21	6	3	+	+	-	+	m	m	-	-	m
22	7	3	+	+	-	+	-	m	-	-	m

[1]Limit for positive recording: Copynr ≥10. [2]Collected immediately after trimming (time point B). [3]Norwegian dairy herds. [4]All possible 9 locations: The grinder (disc/cutting blades (1), shield (2), handle (3)), gloves (4), hoof knife (5), hoof tester (6), chute (footrest (7), cuff (8), belly belt (9). [5]m= missing swabs
